# PET/MRI-Evaluated Activation of Brown Adipose Tissue via Cold Exposure Impacts Lipid Metabolism

**DOI:** 10.3390/metabo12050456

**Published:** 2022-05-19

**Authors:** Katarzyna Miniewska, Katarzyna Maliszewska, Karolina Pietrowska, Joanna Godzień, Łukasz Łabieniec, Małgorzata Mojsak, Adam Krętowski, Michał Ciborowski

**Affiliations:** 1 Metabolomics Laboratory, Clinical Research Centre, Medical University of Bialystok, Marii Sklodowskiej-Curie 24A, 15-276 Bialystok, Poland; karolina.pietrowska@umb.edu.pl (K.P.); joanna.godzien@umb.edu.pl (J.G.); 2 Department of Endocrinology, Diabetology and Internal Medicine, Medical University of Bialystok, Marii Sklodowskiej-Curie 24A, 15-276 Bialystok, Poland; katarzyna.maliszewska@umb.edu.pl (K.M.); adam.kretowski@umb.edu.pl (A.K.); 3 Department of Condensed Matter Physics, University of Bialystok, Konstantego Ciolkowskiego 1L, 15-245 Białystok, Poland; lukaszlabieniec@gmail.com; 4 Independent Laboratory of Molecular Imaging, Medical University of Bialystok, Zurawia 71A, 15-540 Bialystok, Poland; malgorzata.mojsak@umb.edu.pl; 5 Clinical Research Centre, Medical University of Bialystok, Marii Sklodowskiej-Curie 24A, 15-276 Bialystok, Poland

**Keywords:** brown adipose tissue, cold exposure, untargeted metabolomics, plasma lipids

## Abstract

Although brown adipose tissue (BAT) is considered to play a protective role against obesity and type 2 diabetes, the mechanisms of its activation and associations with clinical parameters are not well described. Male adults underwent a 2 h cold exposure (CE) to activate BAT and, based on the results of PET/MRI performed after the CE, were divided into BAT(+) and BAT(−) groups. During the CE procedure, blood samples were collected and alterations in plasma metabolome in both groups were investigated using LC-MS. Additionally, associations between clinical factors and BAT were examined. Moreover, levels of glucose, insulin, leptin, TNF-α, FGF21, and FABP4 were assessed in serum samples. In the BAT(+) group, levels of LPC(17:0), LPE(20:4), LPE(22:4), LPE(22:6), DHA, linoleic acid, and oleic acid increased during CE, whereas levels of sphinganine-phosphate and sphingosine-1-phosphate decreased. Levels of LPE(O-18:0), 9-HpODE, and oleic acid were elevated, while the level of LPE(20:5) was reduced in BAT(+) compared to BAT(−) subjects. AUCs of LPC(18:2), LPC(O-18:2)/LPC(P-18:1), and SM(d32:2) negatively correlated with BAT. In the BAT(+) group, the concentration of FABP4 during and after CE was decreased compared to the basal level. No alterations were observed in the BAT(−) group. In conclusion, using untargeted metabolomics, we proved that the plasma metabolome is affected by cold-induced BAT activation.

## 1. Introduction

As reported by Eurostat, in 2019, 53% of adult Europeans were overweight, one third of which were obese [1]. Obesity is a key factor in the development of insulin resistance (IR), which occurs ten to twenty years before type 2 diabetes (T2DM) onset [2]. The risk of T2DM can be diminished with the reduction of IR and IR decreases with weight loss. Although a change in lifestyle (improvement of nutritional habits and physical activity) is usually effective and individuals lose weight, it is difficult for them to accept a new lifestyle and, unfortunately, they regain weight [3]. On this account, new therapeutic strategies for fighting obesity, and thus IR, are needed. One of the potential targets is brown adipose tissue (BAT) and its role in metabolic processes [4]. BAT is of great importance in heat production via non-shivering thermogenesis, regulated by the activity of uncoupling protein 1 (UCP1), occurring in the inner mitochondrial membrane [5]. Protons released in the lipolysis of triradylglycerols (TG) are transferred inside mitochondria, bypassing the oxidative phosphorylation pathway and subsequent ATP synthesis, which results in energy release in the form of heat [5,6]. For many years, scientists believed that BAT occurred only in infants and it disappeared with age. However, in 2003, accidental findings were published, changing this concept, as it was suggested that regions of higher glucose uptake detected during positron emission tomography (PET) might in fact be BAT [7,8]. Since then, the method used for BAT determination in humans has been PET coupled with either computer tomography (CT) or magnetic resonance (MR). In recent years, mass spectrometry (MS) has become widely applied in the measurement of numerous compounds, particularly small molecules [9]. A combination of MS and separation techniques, especially liquid or gas chromatography (LC or GC, respectively), allows the measurement of even thousands of compounds in biological samples. Since the cold-induced alterations in the plasma metabolome associated with BAT are not well described, untargeted metabolomics seems to be a promising tool in elucidating this scientific problem. With the emerging expectations of BAT as a tool to fight obesity and T2DM, it is crucial to understand the mechanisms of its activation and seek associations between BAT and clinical parameters describing IR. Therefore, the aim of this study was to activate BAT via cold exposure (CE) and to investigate alterations in the plasma metabolome during CE in individuals possessing BAT in comparison to those lacking it, as well as to examine associations between clinical factors and BAT volume and activity.

## 2. Results

### 2.1. Study Group Characteristics

**Figure 1:**
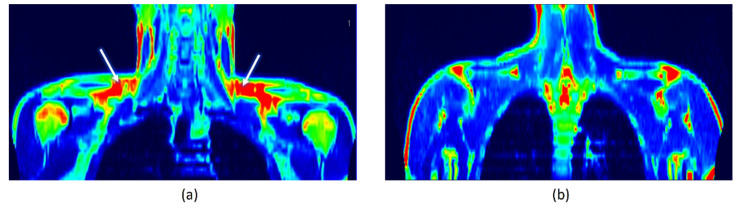
(**a**) A PET/MRI scan of BAT(+) individual. White arrows show BAT areas in supraclavicular regions. (**b**) A PET/MRI scan of BAT(−) individual.

The activation of BAT was observed in 17 subjects, with the median of activity of 29.92 µmol × (100 g^−1^) × min^−1^ and the median of volume of 15,280 mm^3^. Individuals with detectable BAT were classified as the BAT(+) group, whereas those who did not demonstrate the presence of BAT composed the BAT(−) group (Figure 1a,b, respectively).

The BAT(+) and BAT(−) groups did not differ in terms of age, BMI, body composition, or oral glucose tolerance test (OGTT) results. More detailed characteristics are presented in Table 1.

### 2.2. Metabolic Fingerprinting of Plasma Samples

The untargeted analysis of plasma samples obtained during CE was conducted and two datasets (one from positive and the other from negative ion mode) were generated. They subsequently underwent a filtering and QA procedure and, as a result, 1540 metabolic features (in total from positive and negative ion mode) were obtained.

**Figure 2:**
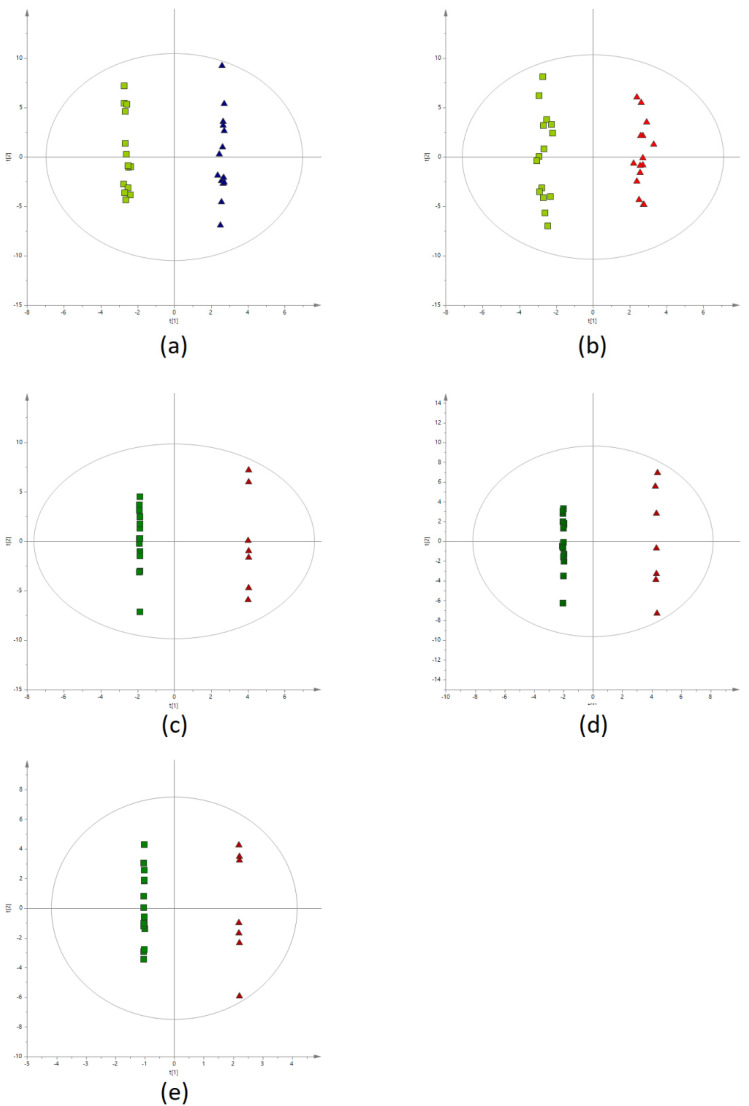
OPLS-DA score plots. (**a**) The classification of BAT(+) samples collected before (green squares) and after 60 min of CE (blue triangles) (negative ion mode, R^2^ = 0.998, Q^2^ = 0.472). (**b**) The classification of BAT(+) samples collected before (green squares) and after 120 min of CE (red triangles) (negative ion mode, R^2^ = 0.991, Q^2^ = 0.505). (**c**) The discrimination of BAT(+) (green squares) and BAT(−) (red triangles) samples collected after 60 min of CE (negative ion mode, R^2^ = 1, Q^2^ = 0.568). (**d**) The discrimination of BAT(+) (green squares) and BAT(−) (red triangles) samples collected after 120 min of CE (negative ion mode, R^2^ = 1, Q^2^ = 0.542). (**e**) The discrimination of BAT(+) (green squares) and BAT(−) (red triangles) samples collected after 120 min of CE (positive ion mode, R^2^ = 1, Q^2^ = 0.609).

Multivariate statistics (OPLS-DA models) were used to examine the discrimination between the samples from different time points (0′ vs. 60′ and 0′ vs. 120′) for BAT(+) and BAT(−) data separately, as well as between BAT(+) and BAT(−) samples in each time point (0′, 60′, and 120′). In case of time point comparisons, it was not possible to build models with acceptable quality for BAT(−) data recorded in negative ion mode. On the contrary, models for BAT(+) revealed good separation between the samples from 0′ and 60′ of CE, as well as samples from 0′ and 120′ (Figure 2a,b, respectively). In terms of positive ion mode, building the models with accepted quality was not possible. For the comparisons of BAT(+) and BAT(−) metabolites’ levels at particular time points, two satisfactory models were created based on negative ion mode data obtained for samples collected after 60 and 120 min of CE (Figure 2c,d, respectively). In positive ion mode data, the only satisfactory model was created for samples collected after 120 min of CE (Figure 2e).

**Figure 3:**
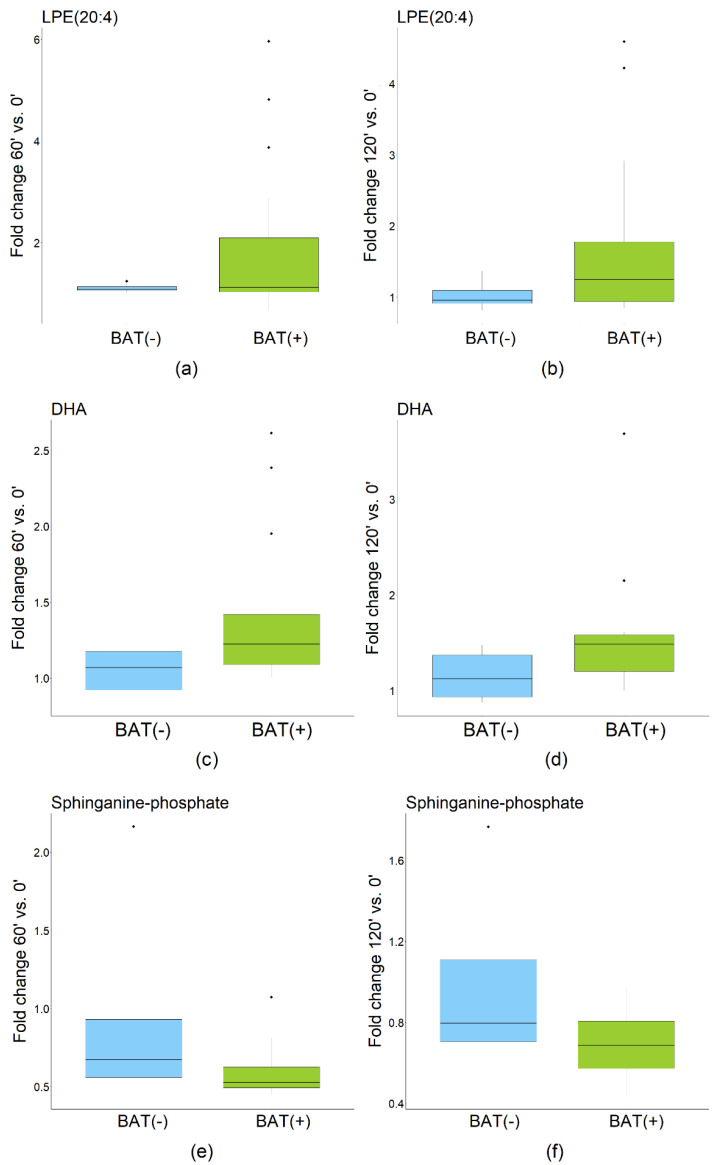
Fold change (FC) of most significant metabolites in BAT(+) and BAT(−) group. (**a**) Fold change of LPE(20:4) after 60 min of CE; (**b**) Fold change of LPE(20:4) after 120 min of CE; (**c**) Fold change of DHA after 60 min of CE; (**d**) Fold change of DHA after 120 min of CE; (**e**) Fold change of sphinganine-phosphate after 60 min of CE; (**f**) Fold change of sphinganine-phosphate after 120 min of CE. The dots indicate outliers.

Statistical analysis revealed 67 and 92 metabolites discriminating samples collected before CE from those collected after 60 and 120 min of CE, respectively. These differences were statistically significant in the BAT(+) but not in the BAT(−) group. After annotations and removal of redundancies, we obtained 13 and 21 metabolites for each comparison, respectively. Metabolites were annotated with L2 or L2/L3 confidence level, according to the classification system proposed by Schrimpe-Rutledge et al. [10], and are presented in Table S1 (available in Supplementary Materials file). In the BAT(+) group, the following compounds were found to significantly increase during CE: LPC(17:0), LPE(20:4), LPE(22:4), LPE(22:6), DHA, linoleic acid, and oleic acid. Meanwhile, the levels of sphinganine-phosphate, and sphingosine-1-phosphate significantly decreased during CE. Metabolites with the highest fold change after 60 or 120 min of CE are presented in Figure 3.

**Figure 4:**
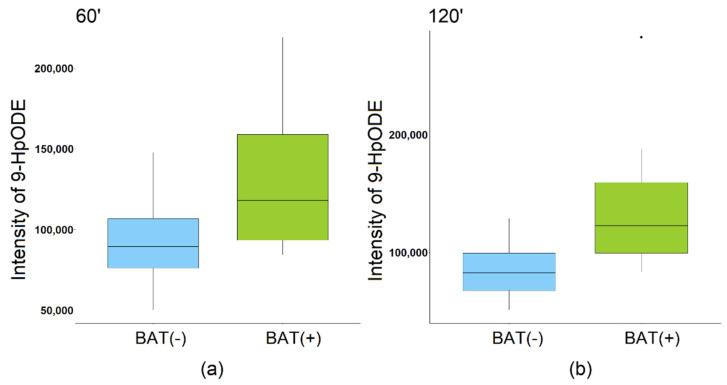
Intensity of 9-HpODE: (**a**) 60th minute of CE; (**b**) 120th minute of CE.

Statistical analyses revealed 86 and 117 metabolic features discriminating the BAT(+) group from the BAT(−) group after 60 and 120 min of CE, respectively (negative ion mode). In terms of positive ion mode, 30 metabolic features were found to discriminate the BAT(+) group from BAT(−) after 120 min of CE. After annotations and removal of redundancies, we obtained 13 metabolites discriminating the studied groups in the 60th minute and 21 in the 120th minute (combining positive and negative ion mode). Again, metabolites were annotated with L2 or L2/L3 confidence level and are presented in Table S2. We observed that the levels of LPE(O-18:0), 9-HpODE, and oleic acid were significantly higher in BAT(+) in comparison to the BAT(−) group, while the level of LPE(20:5) was found to be reduced in BAT(+) in comparison to the BAT(−) group. The intensity of 9-HpODE, which exhibited the highest differences between the BAT(+) and BAT(−) groups after both 60 and 120 min of CE, is presented in Figure 4.

Analysis of Spearman’s correlation between the area under the curve (AUC) of metabolite levels during CE and BAT activity and volume was also performed. A total of 42 and 50 metabolic features were found to correlate with the activity and volume of BAT, respectively (negative ion mode). In positive ion mode, 25 and 12 metabolic features were correlated with the activity and volume of BAT, respectively. After annotation, removal of redundancies, and combining data from positive and negative ion mode, 27 and 33 metabolites were found correlated with the activity and volume of BAT, respectively.

Correlation coefficients and *p*-values are presented in Table 2.

### 2.3. Pathway Analysis

**Figure 5:**
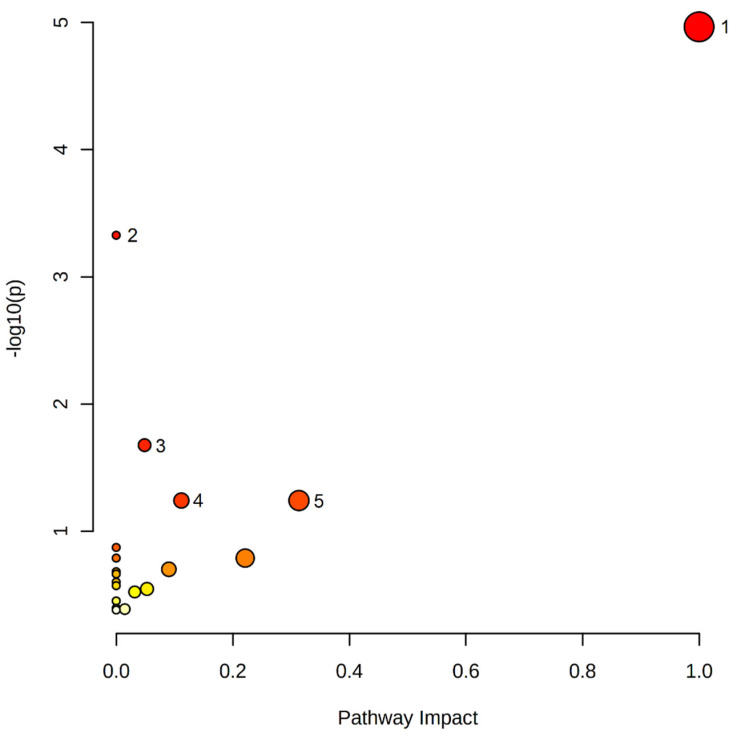
The pathway analysis of all significant metabolites: 1—Linoleic acid metabolism; 2—Biosynthesis of unsaturated fatty acids; 3—Sphingolipid metabolism; 4—Glycerophospholipid metabolism; 5—Arachidonic acid metabolism.

Forty-six metabolites were included in the pathway analysis. These metabolites were mapped into 20 metabolic pathways. The results of the pathway analysis are shown in Figure 5.

**Table 2:** Plasma metabolites correlating with the activity and/or the volume of BAT.

Metabolite	BAT Volume		BAT Activity	
	Correlation Coefficient	*p*-Value	Correlation Coefficient	*p*-Value
L2 annotations				
A, Phospholipids				
LPC(14:0)	−0.45	0.04	−0.48	0.02
LPC(15:0)	−0.50	0.02	−0.42	0.05
LPC(16:0)	−0.34	0.1	−0.46	0.03
LPC(16:1) sn-2	−0.55	0.009	−0.41	0.06
LPC(16:1) sn-1	−0.42	0.05	−0.61	0.003
LPC(17:0)	−0.60	0.003	−0.48	0.02
LPC(17:1)	−0.43	0.04	−0.31	0.2
LPC(18:0) sn-2	−0.58	0.005	−0.50	0.02
LPC(18:0) sn-1	−0.59	0.004	−0.57	0.006
LPC(18:1)	−0.50	0.02	−0.36	0.1
LPC(18:2)	−0.65	0.0009	−0.46	0.03
LPC(19:0)	−0.63	0.002	−0.58	0.005
LPC(19:1)	−0.47	0.03	−0.30	0.2
LPC(20:0)	−0.30	0.2	−0.51	0.01
LPC(20:2)	−0.62	0.002	−0.51	0.02
LPC(20:4)	−0.62	0.002	−0.43	0.05
LPC(O-18:1)/LPC(P-18:0) sn-2	−0.58	0.005	−0.47	0.03
LPC(O-18:1)/LPC(P-18:0) sn-1	−0.47	0.03	−0.35	0.1
LPC(O-18:2)/LPC(P-18:1)	−0.67	0.0006	−0.43	0.05
LPC(16:0)-OH	−0.53	0.01	−0.44	0.04
LPE(18:0)	−0.50	0.02	−0.50	0.02
LPE(O-20:1)/LPE(P-20:0)	−0.49	0.02	−0.45	0.03
LPI(18:0)	−0.43	0.05	−0.24	0.3
PC(16:0_18:3)	−0.54	0.009	−0.63	0.002
PC(16:0_22:6)	−0.46	0.03	−0.34	0.1
PC(36:4)	−0.39	0.07	−0.54	0.009
PC(36:5)	−0.48	0.02	−0.62	0.002
PC(38:5)	−0.50	0.02	−0.44	0.04
PC(O-16:1_18:2)/ PC(P-16:0_18:2)	−0.43	0.04	−0.35	0.1
SM(d32:2)	−0.61	0.003	−0.65	0.001
SM(d35:2)	−0.53	0.01	−0.40	0.06
B, Other metabolites				
Histidine	−0.45	0.04	−0.54	0.009
L2/L3 annotations				
Bilirubin	−0.44	0.04	−0.47	0.03
DHA	0.52	0.01	0.48	0.02
Hexacosanedioic acid	−0.39	0.07	−0.50	0.02
FA 12:4;O3	−0.59	0.003	−0.51	0.02
ST 19:1;O2;S (dihydrotestosterone sulfate)	−0.67	0.0007	−0.51	0.02

LPC—lysophosphatidylcholine; LPE—lysophosphatidylethanolamine; LPI—lysophosphatidylinositol; PC—phosphatidylcholine; SM—sphingomyelin; DHA—docosahexaenoic acid; FA—fatty acid.

### 2.4. Additional Analyses

**Figure 6:**
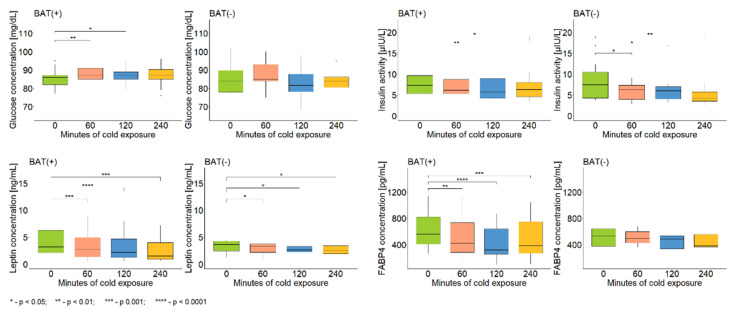
Differences in the level of glucose, insulin, leptin, and FABP4 during and after CE. The statistical differences were analyzed by Wilcoxon signed-rank test.

Additionally to metabolomics analysis, the assessment of the level of glucose, insulin, and several proteins (listed in Table 3) was performed. The concentration of glucose was measured in plasma samples, whereas other parameters were assessed in serum samples. The measurements were conducted in samples collected before, after 60 min of CE, after 120 min of CE, and after 240 min of CE (i.e., 120 min after the end of CE). The results are presented in Table 3 (where median fold change and *p*-value of Wilcoxon signed-rank test are shown) as well as in Figure 6. Regardless of the BAT presence, the glucose concentration was stable during the whole procedure of CE and 120 min after its end, whereas insulin and leptin levels were decreased in comparison to the initial levels. The concentration of fatty acid binding protein 4 (FABP4) during and after CE was decreased, compared to its level at the beginning of CE, but only in the BAT(+) group, as no alterations were noticed in the BAT(−) group. No differences in the concentration of tumor necrosis factor α (TNF-α) and fibroblast growth factor 21 (FGF21), in neither BAT(+) nor BAT(−), were observed.

## 3. Discussion

The aim of this paper was to find metabolites associated with the activity and volume of BAT. Therefore, participants underwent the procedure of CE to activate BAT, and afterwards, a PET/MRI scan was performed to assess its activity and volume. Metabolomics analysis was conducted to understand the influence of this tissue on small molecules’ regulation. We focused not only on metabolites significantly changing after CE, but also discriminating BAT(+) and BAT(−) individuals. In the BAT(+) group, the following compounds were found to significantly increase during CE: LPC(17:0), LPE(20:4), LPE(22:4), LPE(22:6), DHA, linoleic acid, and oleic acid. Meanwhile, the levels of sphinganine-phosphate and sphingosine-1-phosphate significantly decreased during CE. We also obtained significant differences in metabolite levels in the 60th and 120th minute of CE between the BAT(+) and BAT(−) groups, and the levels of LPE(O-18:0), 9-HpODE, and oleic acid were found significantly higher in BAT(+) in comparison to the BAT(−) group, while the level of LPE(20:5) was found to be reduced in BAT(+) in comparison to the BAT(−) group. The AUCs of the following metabolites were negatively correlated with the activity and volume of BAT: LPC(18:2), LPC(O-18:2)/LPC(P-18:1), and SM(d32:2). Additionally, pathway analysis was performed for statistically significant metabolites. The results of pathway analysis (Figure 5) revealed that linoleic acid metabolism was the most affected. Other involved pathways included the biosynthesis of unsaturated fatty acids, sphingolipid metabolism, glycerophospholipid metabolism, and arachidonic acid metabolism.

The association between the serum/plasma metabolome and BAT in humans has been investigated but knowledge on this topic is still very limited. Boon et al. [11] conducted a study in which healthy lean adults underwent the procedure of CE and subsequently had the activity and volume of BAT assessed by 18F-FDG PET/CT. They discovered that fasting levels of LPC(16:0), LPC(16:1), and PC(32:1) positively correlated with BAT activity and/or volume; additionally, the level of PC(32:1) was increased after 120 min of CE (in comparison to its fasting level). In our study, we also observed an association between BAT and LPC(16:0) and LPC(16:1), although the correlation was negative. It has to be considered that we examined the AUC of these metabolites (reflecting their alterations during CE), which could have resulted in the different findings. Xiang et al. [12] aimed to identify plasma non-esterified fatty acids (NEFAs) and oxylipins potentially associated with cold-activated BAT in humans. They discovered that levels of 33 NEFA species were elevated during CE and the largest alterations were observed in the levels of NEFA C14:1 and NEFA C14:2. Furthermore, there was a positive correlation between DHA and EPA baseline level and BAT activity, as well as between the level of 18 NEFA species after CE and BAT activity. Additionally, Xiang et al. noted an increase in the levels of 11 oxylipins species during CE, as well as a positive correlation between BAT volume and the concentration change of 14(15)-EpETrE, 15-HETE, and 15-HETrE. These results are in line with our findings as we observed an increase in DHA level during CE, as well as a positive correlation between its AUC and BAT activity and volume. In terms of oxylipins, we discovered that the level of 9-HpODE after 120 min of CE was significantly higher in BAT(+) individuals. Kovaničová et al. [13] examined changes in the plasma metabolome in humans after 10–15 min of ice-water swimming and found changes in 70 metabolites, the majority of which were amino acids. In the presented study, we did not observe changes in amino acids in response to CE; however, it should be noted that the procedures of CE were significantly different. The involvement of fatty acids and oxylipins in BAT activation was also noted in animal studies. Bargut et al. [14] examined the influence of EPA and DHA supplementation on the markers of browning in WAT, and of thermogenesis in BAT in mice fed with a high-fructose diet. The results showed the enhancement of the expression of browning genes in subcutaneous WAT and thermogenic genes in BAT. Félix-Soriano et al. [15] discovered that a high-fat diet with DHA supplementation restored UCP1 expression in BAT. Lynes et al. [16] observed that the level 12,13-diHOME was elevated in the plasma of humans and mice exposed to cold. All these findings stand in compliance with our observations: the increase in DHA, linoleic, and oleic acid levels during CE, as well as the elevated level of 9-HpODE after 120 min of CE in BAT(+) individuals.

With the exception of fatty acids and related metabolites, our study revealed the association between sphingolipids and sphingoid base lipids and BAT. We observed a decline in the levels of sphinganine-phosphate and sphingosine-1-phosphate during CE in the BAT(+) group, as well as a negative correlation between the AUC of SM(d32:2) and BAT activity and volume. To the best of our knowledge, this is the first study identifying these metabolites in the context of BAT activity and volume in humans, although it is known that sphingolipids and sphingoid base lipids are associated with obesity and IR. Błachnio-Zabielska et al. [17] observed increased levels of sphingosine-1-phosphate in the adipose tissue of obese subjects and a negative correlation between HOMA-IR and total ceramide content in fat tissue. Furthermore, in another study from the same group [18], it was discovered that adipocyte sphinganine, sphingosine, and sphingosine-1-phosphate content was higher in obese compared to lean individuals. Ravichandran et al. [19] examined the role of sphingosine kinase 2 (Sphk2) (involved in the phosphorylation of sphingosine to form sphingosine-1-phosphate) in glucose and lipid metabolism. The authors found that deletion of the Sphk2 gene in mice resulted in protection against age-related weight gain and IR, and knocked-out mice exhibited lower fat mass but greater lean mass as well as increased energy expenditure in comparison to the wild type. Regarding the association between sphingolipids and BAT, Christoffersen et al. [20] suggested that apolipoprotein M (Apom) is involved in BAT activity control, which is mediated by sphingosine-1-phosphate. Apom-/- mice fed with a high-fat diet had lower body weight and better glucose tolerance than wild-type mice. In addition, the authors analyzed the influence of a functional sphingosine-1-phosphate receptor antagonist (FTY720) and discovered an increase in BAT weight in wild-type mice but observed no changes in knocked-out mice. Rajakumari et al. [21] examined the effects of β3-adrenergic stimulation on the mitochondrial lipidome of adipose tissue. It was observed that WAT mitochondria had increased SM levels in comparison to BAT, and as a result of β3-adrenergic stimulation, the level of SM(16:0), SM(22:0), and SM(24:1) was decreased in WAT, but not in BAT mitochondria. As we observed the inverse association between sphingolipids and sphingoid base lipids and BAT, and the abovementioned findings show that the decline in sphingolipids level, especially sphingosine-1-phosphate, may play a crucial role in the protective action of BAT in IR and obesity development, more research is needed to discover its exact mechanism.

In addition, to better understand BAT physiology, we measured levels of glucose, insulin, and several serum proteins and examined their alterations during CE. The concentration of glucose was steady during CE, whereas the activity of insulin decreased during this procedure, which is in line with many data claiming that CE improves glucose homeostasis and insulin sensitivity [22,23,24]. In terms of leptin, our findings also confirmed the literature data, showing a drop in its concentration in response to CE [25,26,27].

Because of the fact that several of observed metabolites were fatty acids, we decided to further examine this topic and assess the level of FABP4 before, during, and 2 h after CE. Interestingly, we observed a decrease in FABP4 concentration in serum during CE, but only in the BAT(+) group. Yamamoto et al. [28] analyzed the expression level of 10 FABP isoforms in the BAT of rats and discovered that FABP4 was the most abundantly expressed, while FABP3 and FABP5 were also, but less, expressed. Furthermore, the authors examined changes in these proteins’ expression in response to CE and found that FABP3 and FABP5 levels were elevated but the FABP4 level remained stable. We noted a decline in FABP4 level during CE, but it is crucial to remember that we measured the FABP4 concentration in serum samples, whereas Yamamoto et al. analyzed tissue samples. Vergnes et al. [29] discovered that although the FABP4 gene was most abundantly expressed in BAT, it was the FABP3 gene whose level was increased during CE, while FABP4 remained unaltered. Again, a possible explanation for our results being contrary is the difference in biological material analyzed. Shu et al. [30] investigated the role of FABP4 in cold-induced thermogenesis in mice and observed an increase in the FABP4 level in serum after 1 h of CE and a further decline to a basal level. This is partially contrary to our findings, as we only observed the decline in its concentration. However, the study of Shu et al. was performed on an animal model and it is possible that the dynamics of the FABP4 level during CE are different in humans. These findings and the lack of studies on FABPs’ role in BAT activation in humans suggest the need for deeper investigation on this topic.

Our study did not avoid certain limitations and the small study group is one of them. However, one should note the cost of the PET/MRI test and the fact that the procedure of CE could only be performed during cold seasons. Furthermore, we could use our PET/MRI center only twice a week to perform this study, which additionally reduced our ability to examine more participants. Another limitation is the restriction in terms of the sex of participants. The body composition of women may be associated with the day of the menstruation cycle [31]; therefore, all potential female participants should have been examined in the same cycle phase. Given the above-described limitations, collecting a homogenous group of women would be unusually difficult.

## 4. Materials and Methods

### 4.1. Study Group

We performed the CE procedure and PET/MR scanning in a group of 37 healthy adult individuals. All procedures were performed from October to April of 2016–2018. It turned out that 17 of them possessed BAT, and to make the study group as coherent as possible, other participants matched in terms of age and BMI were chosen as a control group. Finally, the group consisted of 25 healthy, non-smoking Caucasian males (n = 17 in study group, and n = 8 in control group). The exclusion criteria were any acute or chronic diseases (e.g., asthma, hypo- or hyperthyroidism, cardiovascular diseases), or taking any medication (e.g., beta-blockers or statins). Clinical characteristics are presented in Table 1 in the Results section.

### 4.2. Screening Visit

The screening visit comprised basic laboratory tests (liver enzymes, creatinine, C-reactive protein, lipid profile, TSH, and glycated hemoglobin (HbA1C)) as well as OGTT performed according to WHO guidelines with the ingestion of 75 g glucose load (results can be seen in Table 1). Basic laboratory tests were performed on fasting samples (whole blood for HbA1C, serum for other parameters), whereas glucose and insulin levels (in plasma or serum, respectively) were assessed at the beginning of OGTT (before glucose ingestion) and 30, 60, and 120 min after glucose ingestion. Glucose concentration was measured with the enzymatic hexokinase method using the Cobas c111 analyzer (Roche Diagnostics, Basel, Switzerland) and insulin activity was evaluated with the immunoradiometric assay (DIAsource, Nivelles, Belgium).

During the screening visit, all subjects also underwent body composition analyses using two methods: bioimpedance (InBody 720, Biospace, Seul, Korea) and dual energy X-ray absorption (DXA) (Lunar iDXA, GE Healthcare, Chicago, IL, USA). The following parameters were measured: adipose tissue mass and percentage (AT and AT%, respectively), skeletal muscle mass and percentage (SMM and SMM%, respectively), visceral adipose tissue (VAT) volume and mass.

### 4.3. CE and PET-MR Scanning

The second visit consisted of two hours of CE followed by PET-MR scanning, as described previously [32]. A personalized procedure of cooling was applied to activate BAT. Briefly, individuals lay on a bed between two water-filled blankets (Blanketroll III, Cincinnati Sub-Zero, Cincinnati, OH, USA). The initial water temperature was 22 °C and it was gradually decreased by 2 °C every 10 min until shivering was observed or reported by subjects, and it was then increased by 4 °C. Subjects had their blood collected before and 60 and 120 min after the beginning of the procedure of cooling.

Afterwards, participants underwent a whole-body PET-MR scan (Biograph mMR 3T, Siemens Healthcare, Erlangen, Germany) with an 18F-fluorodeoxyglucose injection (18F-FDG) (4 MBq/kg of body mass). PET-MR results were analyzed using the Carimas software, developed at the Turku PET Centre in Finland. To assess the activity of BAT, the influx rate constant (Ki) of 18F-FGD was determined using the Gjedde-Patlak model. A lumped constant (LC) value of 1.14 was used for calculations [33]. The glucose uptake rate was calculated as follows: plasma glucose concentration × Ki × LC^−1^. The activation of BAT was defined as a glucose uptake rate higher than 2.0 µmol × (100 g^−1^) × min^−1^. Individuals were classified as BAT-positive (BAT(+)) or BAT-negative (BAT(−)), based on detected or non-detected BAT, respectively.

### 4.4. Plasma Metabolic Fingerprinting

Plasma metabolic fingerprinting was performed on plasma samples collected during CE. Whole blood was collected into vacuum system tubes with sodium EDTA as an anticoagulant, and plasma was obtained by immediate centrifugation. Samples were stored at −80 °C until analysis. Analyses were conducted as described previously [34]. Briefly, samples were thawed on ice on the day of analysis. Protein precipitation and metabolite extraction were performed with methanol/ethanol (1:1) mixture containing 1 ppm zomepirac as internal standard. A mixture of an equal volume of all samples was prepared to obtain quality control (QC) samples. All samples were randomly analyzed by an LC-MS system (Agilent Technologies, Santa Clara, CA, USA). Analyses were performed in positive (ESI+) and negative (ESI-) ion modes. The raw data collected by the analytical instrumentation were cleaned of background noise and unrelated ions by the molecular feature extraction (MFE) tool in Mass Hunter Qualitative Analysis Software (B.07.00, Agilent, Santa Clara, CA, USA). Sample alignment and data filtering were performed using Mass Profiler Professional 12.6.1 (Agilent, Santa Clara, CA, USA). Parameters applied for the alignment were 1% for RT and 15 ppm for the mass variation. In the quality assurance (QA) procedure, metabolic features detected in >50% in QC samples with the coefficient of variation (CV) <20% were kept for further data treatment. Metabolite annotation was performed based on tandem mass spectroscopy (MS/MS) fragmentation. The identity of metabolites was confirmed (1) by matching the experimental MS/MS spectra to MS/MS spectra from databases (i.e., METLIN, KEGG, LIPIDMAPS, and HMDB) or (2) based on the fragmentation pattern. Characteristic fragments of identified metabolites are presented in Table S3.

### 4.5. Serum Protein Measurement

The concentration of the following proteins was assessed in serum samples collected during CE: FGF21, TNF-α, FABP4, and leptin. Whole blood was collected into vacuum system tubes with gel clotting activator. Tubes were placed vertically at room temperature for 60 min to form a clot. Afterwards, tubes were centrifuged to obtain serum and samples were stored at −80 °C until analysis. Concentration of FGF21, TNF-α, and FABP4 was determined using a dedicated enzyme-linked immunosorbent assay kit (Quantikine ELISA, R&D Systems, Minneapolis, MN, USA) according to the manufacturer’s instructions. Leptin concentration was determined using an enzyme-linked immunosorbent assay kit (BioVendor, Brno, Czech Republic).

### 4.6. Statistical Analysis

Statistical analysis of the clinical, body composition, and serum protein data consisted of the Wilcoxon signed-rank test for the comparison of paired data, the Mann–Whitney U test for estimation of differences between BAT(+) and BAT(−) groups (calculations were prepared in R (version 4.0.5, https://www.R-project.org/, accessed on 6 April 2021)), and Spearman’s correlation for the analysis of numerical variables (performed by in-house built script for MATLAB (2018b, MathWorks, Natick, MA, USA)).

Multivariate statistics were used to evaluate the quality of metabolomics data by checking the location of the QC samples on principal component analysis (PCA) plots and to observe sample classification on orthogonal projections to latent structures discriminant analysis (OPLS-DA) plots. The OPLS-DAs were performed to examine the differences between BAT(+) and BAT(−) groups, as well as the alterations between particular time points. Statistically significant metabolites were chosen based on predictive loading values (p(corr)) and variable influence on projection (VIP). Multivariate calculations and plots were performed by using SIMCA−P + 13.0.3.0 (Umetrics, Umea, Sweden). Additionally, AUCs of metabolites during CE were calculated and Spearman’s correlation analysis with clinical parameters was conducted.

Non-parametric methods were chosen due to the small sample size.

### 4.7. Pathway Analysis

Pathway analysis was performed using MetaboAnalyst 5.0 (http://www.metaboanalyst.ca/, accessed on 11 May 2022) [35]. Metabolites that were found to be statistically significant in any comparison were taken into consideration and the analysis was performed based on the Human Metabolome Database identifier. The Kyoto Encyclopedia of Genes and Genomes (KEGG)-based Homo sapiens library was selected for analysis, with a hypergeometric test as the enrichment method and relative-betweenness centrality in pathway typology analysis.

## 5. Conclusions

In conclusion, using untargeted metabolomics, we proved that the plasma metabolome is affected by cold-induced BAT activation. Our results, for the first time, indicated that sphingolipids and sphingoid base lipids are inversely associated with BAT activation as well as its volume and activity. Moreover, we observed a cold-induced decline in the FABP4 concentration only in BAT(+) individuals. The lack of similar studies in humans proves the need for further and more detailed research on this subject.

## Figures and Tables

**Table 1:** Characteristics of BAT(+) and BAT(−) groups.

Parameter		BAT(+) Median (Q1–Q3)	BAT(−) Median (Q1–Q3)	*p*-Value
N		17	8	
BAT activity [µmol × (100 g^−1^) × min^−1^]		29.9 (23.6–37.8)	NA	
BAT volume [mm^3^]		15,280 (1859–31,490)	NA	
Age [years]		24 (23–26.5)	27 (25–32.3)	0.07
BMI [kg/m^2^]		24.5 (22.6–28.7)	26.7 (23.2–31.1)	0.37
% AT [%]		16.5 (13.3–22.0)	22.0 (16.0–25.9)	0.34
% SMM [%]		47.5 (44.2–49.7)	44.5 (42.3–47.0)	0.22
VAT volume [cm^3^]		420 (251–761.5)	775.5 (297.8–1275)	0.29
VAT mass [g]		396 (237.5–718.5)	731.5 (291.8–1203.3)	0.24
HbA1c [mmol/L]		4.9 (4.8–5.2)	5.1 (4.9–5.1)	0.42
OGTT	glc 0′ [μIU/L]	97 (90.8–98.8)	94 (91.5–95.5)	0.25
	AUC glc	183 (171.3–199.5)	175 (166.5–193.5)	0.45
	ins 0′ [μIU/L]	7.6 (6.3–11.8)	8.8 (7.4–10.9)	0.48
	AUC ins	22.4 (14.7–33.4)	26.1 (21.9–51.9)	0.19
Cholesterol [mg/dL]		155 (135–177.5)	174 (148.3–182)	0.40
LDL-cholesterol [mg/dL]		77.6 (65.9–111.5)	101.5 (77.3–106.5)	0.41
HDL-cholesterol [mg/dL]		52 (39–73)	57.5 (47.5–72.5)	0.62
Triglycerides [mg/dL]		86 (46.5–110)	83 (46.3–108.5)	0.98
CRP [mg/L]		0.7 (0.3–2.7)	0.7 (0.4–1.1)	0.93
Creatinine [mg/dL]		1 (0.9–1.1)	0.9 (0.9–1.0)	0.56
ALT [IU/L]		21.8 (16.3–26.6)	17.8 (15.7–28.3)	0.54
AST [IU/L]		20.4 (18.8–25.4)	23.8 (18.3–31.0)	0.66
TSH [μIU/mL]		1.9 (1.5–3.2)	197 (1.4–2.5)	0.51

BAT—brown adipose tissue; BAT(+)—group possessing BAT; BAT(−)—group lacking BAT; %—percentage of adipose tissue in total body mass; % SMM—percentage of skeletal muscle in total body mass; VAT—visceral adipose tissue; OGTT—oral glucose tolerance test; glc 0′—fasting glucose concentration; ins 0′—fasting insulin activity; AUC—area under the curve; HbA1c—glycated hemoglobin; CRP—C-reactive protein; ALT—alanine transaminase; AST—aspartate transaminase; TSH—thyrotropic hormone.

**Table 3:** Median percentage of change and *p*-value of Wilcoxon signed-rank test.

		BAT(−)			BAT(+)		
		60′ vs. 0′	120′ vs. 0′	240′ vs. 0′	60′ vs. 0′	120′ vs. 0′	240′ vs. 0′
Glucose	Change [%]	3.5	−2.4	−1.1	3.4	2.4	1.9
	*p*-value	0.2	0.4	0.7	0.005	0.04	0.1
Insulin	Change [%]	−23.5	−23.4	−35.2	−11.4	−6.6	−16.9
	*p*-value	0.02	0.04	0.008	0.09	0.008	0.04
TNF-α	Change [%]	41.9	59.4	50.6	48.8	12.3	45.9
	*p*-value	0.2	0.04	0.2	0.2	0.3	0.1
FGF21	Change [%]	−27.1	−24.1	−39.0	−25.4	−10.3	−35.1
	*p*-value	0.5	0.5	0.02	0.7	0.3	0.08
Leptin	Change [%]	−12.8	−13.1	−20.7	−15.7	−25.4	−35.4
	*p*-value	0.02	0.02	0.02	0.0007	3 × 10^−5^	0.0001
FABP4	Change [%]	5.1	−9.8	−22.6	−12.9	−30.7	−20.1
	*p*-value	1	0.5	0.5	0.003	5 × 10^−5^	0.0002

BAT(−)—group without detectable brown adipose tissue; BAT(+)—group with detectable brown adipose tissue; TNF-α—tumor necrosis factor α; FGF21—fibroblast growth factor 21; FABP4—fatty acid binding protein 4.

## Data Availability

Data are available upon request in justified cases.

## Supplementary Materials

The following supporting information can be downloaded at: https://www.mdpi.com/article/10.3390/metabo12050456/s1, Table S1. Plasma metabolites discriminating samples collected during cold exposure from the samples collected at the beginning of the procedure. Table S2. Plasma metabolites discriminating BAT(+) group from BAT(−) group after 60 and 120 min of cold exposure. Table S3. Annotation of plasma metabolites significantly associated with BAT activation. Pathway analysis report.
