# Supplementary material for: PET/MRI-Evaluated Activation of Brown Adipose Tissue via Cold Exposure Impacts Lipid Metabolism
Source: Metabolites. 2022 May 19;12(5):456. doi: 10.3390/metabo12050456 (PMC9145038; doi:10.3390/metabo12050456)
Supplement: Supplementary file 1 [file metabolites-12-00456-s001.zip › metabolites-1725742-Supplementary materials.pdf]

**Supplementary materials to:**

# PET/MRI-evaluated activation of brown adipose tissue via cold exposure impacts lipid metabolism

**Katarzyna Miniewska[1,*], Katarzyna Maliszewska[2], Karolina Pietrowska[1], Joanna Godzień[1], Łukasz Łabieniec[3], Małgorzata Mojsak[4], Adam Krętowski[2,5], Michał Ciborowski[1,*]**

1 Laboratory of Metabolomics, Clinical Research Centre, Medical University of Bialystok, Marii Sklodowskiej-Curie 24A, 15-276 Bialystok, Poland; karolina.pietrowska@umb.edu.pl (K.P.); joanna.godzien@umb.edu.pl (J.G.)
2 Department of Endocrinology, Diabetology and Internal Medicine, Medical University of Bialystok, Marii Sklodowskiej-Curie 24A, 15-276 Bialystok, Poland; katarzyna.maliszewska@umb.edu.pl (K.Ma.); adam.kretowski@umb.edu.pl (A.K.)
3 Department of Condensed Matter Physics, University of Bialystok, Konstantego Ciolkowskiego 1L, 15-245 Białystok, Poland; lukaszlabieniec@gmail.com (Ł.Ł.)
4 Independent Laboratory of Molecular Imaging, Medical University of Bialystok, Zurawia 71A, 15-540, Bialystok, Poland; malgorzata.mojsak@umb.edu.pl (M.M.)
5 Clinical Research Centre, Medical University of Bialystok, Marii Sklodowskiej-Curie 24A, 15-276 Bi-alystok, Poland
* Correspondence: katarzyna.miniewska@umb.edu.pl (K.Mi.); michal.ciborowski@umb.edu.pl (M.C.)

Table S1. Plasma metabolites discriminating samples collected during cold exposure from the samples collected at the beginning of the procedure.

| Metabolite | Percentage of change | | | |
|---|---|---|---|---|
| | 60 minutes of CE vs. before CE | | 120 minutes of CE vs. before CE | |
| | BAT (+) (absolute p(corr); VIP) | BAT (-) (absolute p(corr); VIP) | BAT (+) (absolute p(corr); VIP) | BAT (-) (absolute p(corr); VIP) |
| **L2 annotations** | | | | |
| **A, Phospholipids** | | | | |
| **LPC(16:0) sn-2** | NS | NS | -9.8 (0.4; 1.3) | 2.2 (NS) |
| **LPC(16:0) sn-1** | NS | NS | -9.0 (0.5; 1.4) | 1.7 (NS) |
| **LPC(17:0)** | 61.4 (0.5; 2.4) | 4.7 (NS) | NS | NS |
| **LPC(18:0)** | -27.5 (0.4; 2.6) | -11.9 (NS) | -15.6 (0.5; 1.8) | 10.9 (NS) |
| **LPC(18:2) sn-2** | NS | NS | -15.7 (0.5; 1.9) | -4.7 (NS) |
| **LPC(18:2) sn-1** | NS | NS | -10.8 (0.4; 1.4) | -3.7 (NS) |
| **LPE(16:0)** | NS | NS | 40.4 (0.4; 2.3) | 41.4 (NS) |
| **LPE(20:4)** | 93.2 (0.4; 2.5) | 10.5 (NS) | 18.1 (0.4; 1.5) | 13.1 (NS) |
| **LPE(22:4)** | NS | NS | 34.3 (0.4; 1.9) | 11.6 (NS) |
| **LPE(22:6)** | NS | NS | 29.2 (0.4; 1.8) | -1.4 (NS) |
| **B, Other metabolites** | | | | |
| **Citric acid** | NS | NS | 22.9 (0.4; 1.7) | 26.6 (NS) |
| **Lactic acid** | -26.6 (0.5; 2.7) | -6.8 (NS) | -22.2 (0.5; 2.3) | -9.6 (NS) |
| **Sphinganine-phosphate** | -40.4 (0.8; 4.0) | -10.5 (NS) | -31.9 (0.7; 3.2) | -1.7 (NS) |
| **L2/L3 annotations** | | | | |
| **(d18:2) sphingosine** | -26.6 (0.7; 2.9) | -14.9 (NS) | -17.1 (0.5; 2.0) | 2.9 (NS) |
| **Arachidonic acid** | 43.6 (0.4; 2.3) | 29.3 (NS) | 68.7 (0.6; 3.2) | 40.7 (NS) |
| **Cholic acid** | -12.9 (0.4; 3.2) | 35.4 (NS) | -58.3 (0.7; 5.2) | -31.2 (NS) |
| **DHA** | 42.2 (0.4; 2.2) | 6.1 (NS) | 55.8 (0.5; 2.6) | 15.5 (NS) |
| **Hydroxyoctadecanoic acid (FA 18:0-OH)** | 13.4 (0.5; 1.4) | 11.8 (NS) | 13.1 (0.5; 1.4) | 7.2 (NS) |
| **Linoleic acid** | NS | NS | 37.1 (0.5; 2.3) | 7.9 (NS) |
| **LPA(O-18:3);O** | -32.6 (0.6; 3.1) | -21.5 (NS) | -27.7 (0.6; 2.8) | -22.3 (NS) |
| **Oleic acid** | NS | NS | 28.4 (0.5; 1.8) | 2.6 (NS) |
| **Palmitic acid** | 25.3 (0.5; 2.0) | 16.3 (NS) | 27.0 (0.6; 2.0) | 27.9 (NS) |
| **Sphingosine-1-phosphate** | -32.4 (0.7; 3.4) | -8.8 (NS) | NS | NS |

Table S2. Plasma metabolites discriminating BAT(+) group from BAT(-) group after 60 and 120 minutes of cold exposure.

| Metabolite | Percentage of change | |
|---|---|---|
| | **60 minutes of CE BAT (+) vs. BAT (-) (absolute p(corr); VIP)** | **120 minutes of CE BAT (+) vs. BAT (-) (absolute p(corr); VIP)** |
| **L2 annotations** | | |
| **A, Phospholipids** | | |
| **LPC(16:0) sn-2** | NS | -22.0 (0.5; 1.9) |
| **LPC(16:0) sn-1** | NS | -10.3 (0.5; 1.1) |
| **LPC(16:1)** | -9.9 (0.4; 1.8) | NS |
| **LPC(17:0)** | -11.7 (0.5; 2.0) | NS |
| **LPC(18:0) sn-2** | 34.6 (0.4; 1.4) | -21.2 (0.5; 2.0) |
| **LPC(18:0) sn-1** | -22.1 (0.4; 1.3) | -15.4 (0.4; 1.6) |
| **LPC(18:1)** | -12.6 (0.4; 1.3) | NS |
| **LPC(20:1)** | NS | -22.8 (0.4; 1.8) |
| **LPC(20:2)** | NS | -19.9 (0.4; 1.6) |
| **LPC(20:5)** | NS | -46.3 (0.4; 2.3) |
| **LPC(O-16:0)** | -13.8 (0.4; 1.5) | -18.0 (0.5; 1.6) |
| **LPC(O-18:1) / LPC(P-18:0)** | NS | -25.7 (0.4; 1.8) |
| **LPE(O-16:1) / LPE(P-16:0)** | NS | -26.6 (0.5; 2.2) |
| **LPE(O-18:0)** | 43.4 (0.5; 1.8) | NS |
| **LPE(O-18:1) / LPE(P-18:0)** | NS | -22.4 (0.4; 1.8) |
| **LPE(O-18:2) / LPE(P-18:1)** | NS | -28.3 (0.5; 2.1) |
| **PC(36:3)** | NS | -24.1 (0.6; 2.0) |
| **SM(d35:1)** | NS | 31.2 (0.5; 1.8) |
| **SM(d42:2)** | NS | 24.8 (0.4; 2.0) |
| **B, Other metabolites** | | |
| **9-HpODE** | 66.9 (0.5; 2.2) | 61.0 (0.6; 2.6) |
| **Acetylcarnitine** | NS | 28.5 (0.4; 2.0) |
| **L2/L3 annotations** | | |
| **Hexacosanedioic acid** | NS | -29.3 (0.4; 1.8) |
| **Hydroxyoctadecanoic acid** | 39.4 (0.5; 1.7) | 21.9 (0.6; 1.8) |
| **Linoleic acid** | 37.1 (0.6; 3.4) | NS |
| **Oleic acid** | 19.3 (0.5; 2.5) | 59.7 (0.7; 3.1) |
| **Palmitic acid** | 35.1 (0.6; 2.5) | 29.6 (0.7; 3.6) |

Table S3. Annotation of plasma metabolites significantly associated with BAT activation

| Metabolite | RT (min) | Fragments |
|---|---|---|
| **A, Lipids** | | |
| **LPA(O-18:3);O** | 6.1 | N: 433.235, 152.996, 78.959 |
| **LPC(14:0)** | 5.0 | N: 512.299, 452.279, 227.202, 224.071<br>P: 468.308, 450.297, 184.073, 166.034, 125.000, 104.107 |
| **LPC(15:0)** | 5.2 | N: 526.315, 466.294, 241.217, 224.069, 168.041<br>P: 482.323, 464.312, 184.073, 166.061, 124.998, 104.107 |
| **LPC(16:0) sn-2** | 5.5 | N: 540.331, 480.309, 255.233<br>P: 496.340, 184.073 |
| **LPC(16:0) sn-1** | 5.6 | N: 540.331, 480.311, 255.233, 224.070, 168.043<br>P: 496.340, 184.073, 166.062, 125.000, 104.070 |
| **LPC(16:1) sn-2** | 5.0 | N: 538.315, 478.293, 253.217 |
| **LPC(16:1) sn-1** | 5.2 | N: 538.315, 478.294, 253.217, 224.069, 168.043 |
| **LPC(17:0)** | 5.7 | N: 554.344, 494.326, 269.249, 224.070, 168.042 |
| **LPC(17:1)** | 5.4 | N: 552.330, 492.309, 267.233, 224.069 |
| **LPC(18:0) sn-2** | 6.1 | N: 568.362, 508.342, 283.265, 224.070, 168.043 |
| **LPC(18:0) sn-1** | 6.2 | N: 568.362, 508.341, 283.265, 224.067, 168.043 |
| **LPC(18:1)** | 5.8 | N: 566.346, 506.326, 281.249, 224.069, 168.042 |
| **LPC(18:2) sn-2** | 5.3 | N: 564.331, 504.310, 279.233, 168.041<br>P: 520.341, 184.073, 166.062, 125.000, 104.107 |
| **LPC(18:2) sn-1** | 5.4 | N: 564.331, 504.312, 279.233, 224.069, 168.043<br>P: 520.341, 502.328, 184.073, 166.062, 125.000, 104.107 |
| **LPC(19:0)** | 6.5 | N: 582.377, 522.359, 297.279, 224.071 |
| **LPC(19:1)** | 6.0 | N: 580.361, 520.340, 295.265, 224.072 |
| **LPC(20:0)** | 6.8 | N: 596.393, 536.374, 311.296, 224.070 |
| **LPC(20:1)** | 6.3 | N: 594.377, 534.356, 309.279, 224.068 |
| **LPC(20:2)** | 5.9 | N: 592.362, 532.342, 307.264, 224.069 |
| **LPC(20:4)** | 5.4 | N: 578.301, 528.311, 303.233, 242.083, 168.043 |
| **LPC(20:5)** | 5.1 | N: 586.315, 526.296, 301.217, 224.070 |
| **LPC(O-16:0)** | 5.7 | N: 526.351, 466.330, 224.068, 168.044 |
| **LPC(O-18:1) / LPC(P-18:0)** | 5.9 | N: 552.367, 492.346 |

| | | |
|---|---|---|
| **LPC(O-18:2) / LPC(P-18:1)** | 5.9 | N: 550.351, 490.329 |
| **LPC(16:0)-OH** | 5.1 | P: 512.334, 184.073, 146.983, 125.000, 104.107 |
| **LPE(16:0)** | 5.5 | N: 452.278, 255.233, 196.038<br>P: 454.292, 313.234, 282.281 |
| **LPE(18:0)** | 6.2 | N: 480.309, 283.265, 214.049, 196.037 |
| **LPE(20:4)** | 5.4 | N: 500.278, 303.233, 214.049, 196.038 |
| **LPE(22:4)** | 5.8 | N: 528.309, 331.266, 196.040 |
| **LPE(22:6)** | 5.3 | N: 592.265, 524.280, 327.234, 283.244, 196.039 |
| **LPE(O-16:1) / LPE(P-16:0)** | 5.7 | N: 436.283, 196.036, 140.012 |
| **LPE(O-18:0)** | 5.7 | N: 466.329, 196.038, 168.041 |
| **LPE(O-18:1) / LPE(P-18:0)** | 6.3 | N: 464.314, 196.037, 140.012 |
| **LPE(O-18:2) / LPE(P-18:1)** | 5.9 | N: 462.299, 196.038, 140.011 |
| **LPE(O-20:1) / LPE(P-20:0)** | 5.9 | N: 492.346, 168.041 |
| **LPI(18:0)** | 6.8 | N: 599.320, 315.046, 283.264, 241.015, 152.995 |
| **PC(16:0_18:3)** | 9.1 | N: 800.544, 740.524, 277.217, 255.233, 168.042 |
| **PC(16:0_22:6)** | 9.3 | N: 840.532, 790.541, 480.309, 327.232, 283.244, 255.231 |
| **PC(36:3)** | 9.7 | N: 900.506, 116.929 |
| **PC(36:4)** | 9.1 | N: 894.547, 112.985 |
| **PC(36:5)** | 9.0 | N: 892.532, 112.985<br>P: 780.554, 184.073, 166.058, 125.000 |
| **PC(38:5)** | 9.8 | N: 924.506, 116.928 |
| **PC(O-16:1_18:2) /<br>PC(P-16:0_18:2)** | 9.9 | N: 786.565, 726.545, 279.232 |
| **SM(d32:2)** | 7.4 | N: 717.519, 657.498, 168.043 |
| **SM(d35:1)** | 9.0 | N: 761.581, 701.561, 168.043 |
| **SM(d35:2)** | 8.5 | N: 759.565, 699.547, 168.043 |
| **SM(d42:2)** | 7.5 | P: 835.666, 776.593, 652.598, 184.072, 146.982 |
| **B, Other metabolites** | | |
| **(d18:2) sphingosine** | 4.5 | N: 376.225, 78.960 |
| **9-HpODE** | 5.4 | N: 311.223, 293.212, 249.221 |
| **Acetylcarnitine** | 0.2 | P: 204.124, 145.049, 85.028, 60.081 |

| | | |
|---|---|---|
| **Arachidonic acid** | 7.0 | N: 303.233, 259.242, 205.197, 59.013 |
| **Bilirubin** | 4.9 | N: 583.256, 285.125, 253.136 |
| **Cholic acid** | 4.2 | N: 407.280 |
| **DHA** | 6.9 | N: 327.233, 283.243, 229.196, 59.014 |
| **FA 12:4;O3** | 3.5 | N: 239.093, 195.103, 151.113 |
| **Hexacosanedioic acid** | 7.8 | N: 425.363, 407.353, 363.360 |
| **Hydroxyoctadecanoic acid (FA 18:0-OH)** | 7.3 | N: 299.259, 253.254 |
| **Histidine** | 0.2 | N: 154.062, 137.035, 110.072, 93.045, 72.009 |
| **Linoleic acid** | 7.0 | N: 279.233 |
| **Oleic acid** | 7.5 | N: 281.249 |
| **Palmitic acid** | 7.4 | N: 255.233 |
| **Sphingosine-1-phosphate** | 4.9 | N: 446.228, 378.240, 78.959 |
| **ST 19:1;O2;S (dihydrotestosterone sulfate)** | 3.9 | N: 367.158, 96.960 |
